# Retraction: Nassef, Y. et al. The Impact of Aerobic Exercise and Badminton on HDL Cholesterol Levels in Adult Taiwanese. *Nutrients* 2019, *11*, 515

**DOI:** 10.3390/nu12041178

**Published:** 2020-04-22

**Authors:** Nutrients Editorial Office

**Affiliations:** MDPI, St. Alban-Anlage 66, 4052 Basel, Switzerland; nutrients@mdpi.com

The published article [1] has been retracted due to data errors. During a review of the data, the authors found that the overall sample size stated in the abstract, methods, results, and Table 1 were not consistent. Moreover, follow-up data were not excluded during analysis and hence duplicate records were included in Tables 1–4. Although the outcome and conclusions of the study were not affected, the changes to the data were so substantial that it was decided that a correction would not be appropriate, and the paper will therefore be retracted. We apologize for any inconvenience caused by the removal of this article. A new version of the manuscript will be published at a later date.

MDPI is a member of the Committee on Publication Ethics and takes the responsibility to enforce strict ethical policies and standards very seriously. To ensure the addition of only high quality scientific works to the field of scholarly publication, this article [1] is retracted and shall be marked accordingly. We apologize to the readers of *Nutrients*.
